# P-221. Ribotype Ecology and 90-day Mortality Changes after a CDI Diagnostic Change from a PCR-based to a 2-step Diagnostic Algorithm

**DOI:** 10.1093/ofid/ofae631.425

**Published:** 2025-01-29

**Authors:** Ricky Huynh-Phan, Taryn A Eubank, Thanh Le, Anne J Gonzales-Luna, Jinhee Jo, Khurshida Begum, M Jahangir Alam, Todd M Lasco, Mayar Al Mohajer, Kevin W Garey

**Affiliations:** University of Houston, Spring, Texas; University of Houston College of Pharmacy, Houston, Texas; Department of Pharmacy Practice and Translational Research, University of Houston College of Pharmacy, Houston, Texas, USA, Houston, Texas; Department of Pharmacy Practice and Translational Research, University of Houston College of Pharmacy, Houston, TX; University of Houston, Spring, Texas; Department of Pharmacy Practice and Translational Research, University of Houston College of Pharmacy, Houston, Texas, USA, Houston, Texas; Department of Pharmacy Practice and Translational Research, University of Houston College of Pharmacy, Houston, Texas, USA, Houston, Texas; Baylor St. Luke's Medical Center, Houston, Texas; Baylor College of Medicine, Houston, TX; University of Houston, Spring, Texas

## Abstract

**Background:**

Due to detection of *Clostridioides difficile* colonization, many hospitals changed from a PCR-based CDI diagnostic to a two-step diagnostic using a GDH/EIA assay. How this change affected the ecology of diagnostic *C. difficile* strains or patient outcomes is unknown. The purpose of this study was to assess differences in strain ecology and patient outcomes after a CDI-diagnostic change from a PCR-based to a GDH/EIA diagnostic assay.

**Methods:**

Stool samples considered CDI positive by institutional policy were collected from a university-affiliated quaternary care hospital and associated community hospitals from 2016-21. Stool samples were cultured for *C. difficile* and ribotyped by fluorescent PCR. Patient’s health records were reviewed for 30- and 90-day mortality and CDI recurrence. Ribotype distribution and clinical outcomes were compared after the diagnostic change.

**Results:**

The cohort included 418 patients (PCR+: n=315; GDH+/EIA+: n=103). An increased proportion of ribotype (RT) 027 was noted after the change to GDH/EIA and a decreased likelihood of RT014-020 and RT255. An increased likelihood of singletons (PCR ribotypes observed only once) was shown during use of the PCR diagnostic. CDI treatment failures and mortality was higher after implementation of the GDH/EIA diagnostic. Ninety-day mortality was 19.4% using the GDH/EIA diagnostic and 11.1% using the PCR (p=0.030). No differences in CDI recurrence were noted.

**Conclusion:**

*C*. *difficile* ecology and clinical outcomes changed after implementation of a GDH/EIA diagnostic, likely due to PCR detection of colonization.

**Disclosures:**

**Anne J. Gonzales-Luna, PharmD, BCIDP**, Ferring Pharmaceuticals: Advisor/Consultant|Innoviva Specialty Therapeutics: Advisor/Consultant|Merck and Co: Grant/Research Support|Paratek Pharmaceuticals: Grant/Research Support|Seres Therapeutics: Grant/Research Support **Kevin W. Garey, PharmD, MS**, Acurx: Grant/Research Support

